# Developing a Conceptually Equivalent Type 2 Diabetes Risk Score for Indian Gujaratis in the UK

**DOI:** 10.1155/2016/8107108

**Published:** 2016-09-15

**Authors:** Naina Patel, Andrew Willis, Margaret Stone, Shaun Barber, Laura Gray, Melanie Davies, Kamlesh Khunti

**Affiliations:** ^1^Diabetes Research Centre, University of Leicester, Leicester, UK; ^2^Department of Health Sciences, University of Leicester, Leicester, UK

## Abstract

*Aims.* To apply and assess the suitability of a model consisting of commonly used cross-cultural translation methods to achieve a conceptually equivalent Gujarati language version of the Leicester self-assessment type 2 diabetes risk score.* Methods.* Implementation of the model involved multiple stages, including pretesting of the translated risk score by conducting semistructured interviews with a purposive sample of volunteers. Interviews were conducted on an iterative basis to enable findings to inform translation revisions and to elicit volunteers' ability to self-complete and understand the risk score.* Results.* The pretest stage was an essential component involving recruitment of a diverse sample of 18 Gujarati volunteers, many of whom gave detailed suggestions for improving the instructions for the calculation of the risk score and BMI table. Volunteers found the standard and level of Gujarati accessible and helpful in understanding the concept of risk, although many of the volunteers struggled to calculate their BMI.* Conclusions.* This is the first time that a multicomponent translation model has been applied to the translation of a type 2 diabetes risk score into another language. This project provides an invaluable opportunity to share learning about the transferability of this model for translation of self-completed risk scores in other health conditions.

## 1. Introduction

The prevalence of type 2 diabetes (T2DM) and the number of people at high risk of T2DM in the UK have been rising at an increasing rate in recent decades and both are predicted to continue to rise over the next decade [1]. Up to 7 million people in the UK are currently undiagnosed with this condition [2].

Earlier identification and treatment of T2DM can reduce the risk of complications [3, 4]. National consensus guidelines [5–7] relating to the identification of people at high risk of T2DM reflect this evidence.

Guidance recommends a two-staged approach to screening [8] involving the use of a validated risk assessment tool followed by a confirmatory blood test. This can be followed by appropriate referral to evidence based structured lifestyle intervention programmes [5]. In the UK, this approach forms the basis of an innovative national diabetes prevention programme (NHS DPP) currently being piloted, to be implemented nationally in 2016 [9].

Earlier identification of T2DM and those at high T2DM risk is particularly salient for South Asian (SA) populations as their risk of T2DM and associated mortality and morbidity is significantly higher than white Europeans [10]. Due to the increased risk in this population, NICE recommend offering screening at an earlier age of 25 rather than 40 years as for the general population. Although the benefits of NICE recommendations have been acknowledged, concerns have been raised about the capacity of the National Health Service (NHS) to implement these recommendations, particularly in communities characterised by high numbers of people from diverse ethnic groups. This has led to NICE suggesting that non-NHS organisations (faith, voluntary, and community centres) can facilitate access and support for lay people to self-assess their own risk using a validated risk score [5].

The Leicester Self-Assessment Risk Score (LSAS) [11] is an example of a validated risk score that has been developed for use in a multiethnic population for detecting undiagnosed T2DM and those at high risk. It is noninvasive and simple to calculate based on seven demographic variables. The LSAS gives an estimate of T2DM risk and provides advice on what further action should be taken (see Appendix 1 in Supplementary Material available online at http://dx.doi.org/10.1155/2016/8107108).

Language and health literacy levels are significant barriers to the completion of such risk scores in SA populations. These issues were emphasised during early testing of Guajarati and Punjabi forward translated versions of the LSAS. This evaluation was originally undertaken by conducting two separate focus groups with Punjabi Sikhs and Gujarati Hindus. The focus group findings demonstrated a low level of conceptual understanding about the purpose of the LSAS. Participants suggested that accuracy and readability level were low, with some parts being incomprehensible. Additionally, participants advocated that a translated version of the LSAS should be understood by people with a reading and comprehension age of ≥12 years. It was felt that those with a lower reading age would be unable to adequately comprehend and complete the task and would require assistance.

This preliminary work demonstrated the need for further translation and development of the LSAS for completion by non-English speaking individuals. In this paper, we describe how commonly used methods for cross-cultural translation of research instruments [12–14] were used to develop a model that aimed to achieve conceptual and linguistic equivalence [15] for Gujarati speakers with a reading and comprehension age of 12 and above [14]. Due to the demographics of the local population, we initially selected Gujarati as the first language to translate into; this process served as the process through which we developed the translational model.

## 2. Participants and Methods

### 2.1. Methods for Translation of the English LSAS

We received ethical approval for this project from the College Ethics Committee, University of Leicester, UK (ref. 0373), and Local Research Governance approval.

**Figure 1:**
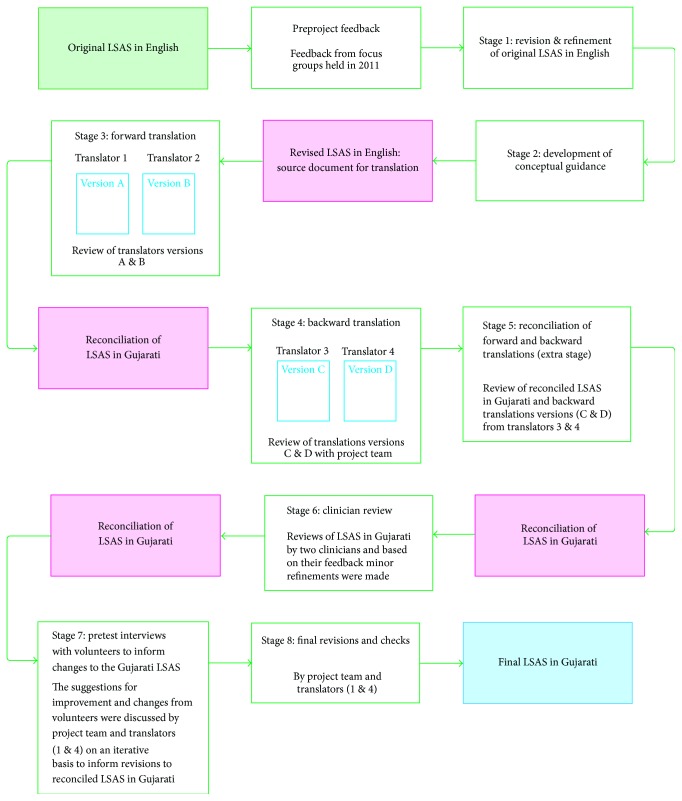


We developed a translation model (Figure 1) based on methods described in cross-cultural translation literature [12–14], including forward and backward translation, clinician review, and pretest interviews with the target population. Below, we provide a description of each stage and the issues that arose.

### 2.2. Recruitment and Selection of Translators

We recruited four experienced translators with a diploma in public service interpreting. This qualification formed part of our selection criteria to ensure a high standard of translation. Two of the translators were already known to the researchers having provided translation for other studies focusing on T2DM. The other two translators were diabetes “naïve” [14]. We assigned two of the translators to stage 3 (one with previous experience and one who was diabetes naïve) and two to stage 4 of the process (Figure 1). Before commencing translation, all translators received project information to help them to contextualise their specific role within the overall project.

### 2.3. Stage One: Revision and Refinement of the English LSAS

The research team made revisions to the LSAS to help clarify the messages in the text using plain English (see Appendix 1). This stage produced a revised version of the English LSAS and it was the source document from which translations were undertaken.

### 2.4. Stage Two: Development of Conceptual Guidance Document for Translators

A conceptual guidance document was developed to specify the intended meaning of each section of text from the LSAS, in order to promote accuracy of translation and conceptual meaning. For example, terms such as BMI have no Gujarati language equivalent; translators were advised to use phonetic translations; they were also advised to retain the use of English words such as diabetes and stroke which are commonly used by UK Gujarati speakers.

### 2.5. Stage Three: Forward Translation

In August 2013, translators 1 and 2 received copies of the source document and conceptual guidance. Once the translations were complete, both translators attended a meeting with the project team to discuss and resolve differences. Examples of issues highlighted during this stage included the use of “everyday spoken language” that was unsuitable for a written document, technical and spelling errors. During the meeting, an agreement was reached about the forward translation using a phonetic translation of the word for “risk,” with its Gujarati equivalent, in English script, in brackets (*jokhem*). The word sugar was phonetically translated with glucose in Gujarati in brackets.

### 2.6. Stage Four: Backward Translation

The reconciled translation was sent to translators 3 and 4 for back translation without the aid of the conceptual guidance. During review by the research team, it was apparent that both translations had captured the meaning of the forward translation, but comparison with the original English highlighted important differences, particularly relating to the complexity of language used and the use of modal verbs (e.g., can and could). Some examples included the use of* “age”* instead of* “getting older,” “consult their GP”* instead of* “talking to their GP,”* and* “you can”* develop T2DM instead of* “could you”* have T2DM?

### 2.7. Stage Five: Reconciliation of Forward and Backward Translations

This additional stage was not in the original project plan but was included to address differences highlighted in the backward translations. It involved three meetings with all the four translators working with the project team. The meetings involved focused discussions about each paragraph of the source document and the forward and backward translations. The discussions were guided by a schedule produced by the research team that detailed differences.

### 2.8. Stage Six: Clinician Review

Two local general practitioners (GPs) who spoke and read Guajarati and used the language in consultations with patients were asked to give their consent and recruited to the study. Both GPs were asked to independently complete a clinical review of the LSAS. This involved use of their knowledge to assess the clinical accuracy of the terms used, as well the appropriateness and accuracy of the content and level of the language used.

### 2.9. Stage 7: Pretest Interviews with Volunteers to Inform Changes to the Gujarati LSAS

Recruitment of Gujarati volunteers took place with the aid of an adult learning organisation. Assistance with recruitment was also given by an Indian Muslim community volunteer who took part in the project as a participant and subsequently helped recruit four additional Indian Muslim participants. The organisation and volunteer were provided with guidance about the eligibility criteria for the project and the purposive sampling strategy, which aimed to recruit a varied group of up to 20 people whose main language was Gujarati. The sample variation was based on factors including age, gender, education level, country of birth, and length of residency in the UK.

A total of 18 Gujarati volunteers who reflected the diversity of the local population in terms of people who were born and educated in India as well as those that migrated from Africa to the UK were recruited to take part.

Before commencing interviews, the researcher (NP) gave each volunteer the participant information sheet (available in English and Gujarati) to read and gave a verbal explanation of the project. Informed consent was recorded for their permission to audio-record the interview, store anonymised interview transcripts electronically, and publish quotations from the transcripts in an anonymised form. Four volunteers arrived in pairs for the interviews; the researcher (NP) checked whether they had any concerns about confidentiality and they were happy to go ahead with taking part in the study. Both pairs completed their LSAS individually but gave feedback together.

During the interviews, participants were asked to self-complete the LSAS, with assistance from the researcher if required. After self-completion, participants were asked to share with the researcher (NP) what they understood from each section of the LSAS and to suggest improvements and changes. Finally, NP facilitated discussions with the aid of a topic guide about volunteers' perception of their risk and views about the LSAS. Qualitative data collection was undertaken on an iterative basis to ensure that volunteers' suggestions could be used to refine and revise the Gujarati LSAS and to document changes suggested for the English LSAS.

Data were collected during 18 interviews, at which point no further suggestions for revising the translation were forthcoming. Volunteers were given a *£*20 store voucher as a token of appreciation for their contributions.

NP transcribed the interviews, simultaneously translating those conducted wholly or partially in Gujarati. The data were organised thematically using framework charts [16] broadly reflecting topic guide themes. Detailed notes were made of volunteers' suggested changes to the text and graphics of the LSAS; these notes informed subsequent discussion between the project team and translators (one from each of the following stages: 3 and 4).

### 2.10. Stage 8: Production of Final Version of the Gujarati LSAS

In response to volunteers' suggestions from stage 7, the project and translators made some additional minor changes to the Gujarati LSAS.

### 2.11. Refining the English Version of the LSAS

Over the course of the project, minor changes to the English LSAS were also made.

## 3. Results

During the translation process, a number of challenges were encountered; examples are provided in Table 1. These challenges were linked to achieving different forms of equivalence (conceptual and linguistic), with some being linked to more than one form. Difficulties with providing satisfactory translations for the terms “risk” and “risk factors,” for example, were linked to conceptual equivalence [15, 17] and also to cultural equivalence, which recognises differences in cultural understandings [15, 17]. Features of the language, including complexity and levels of abstraction [17], were considered during discussions about the education level of the language used, whilst translation and modification of the BMI table involved consideration of operational equivalence [18], related to the need to provide a format which produces equivalent translations. The latter challenge, relating to self-estimation of BMI, proved to be the most challenging to address.

Both GPs felt that the standard of the LSAS translation was very good and was pitched at the appropriate level. They suggested only minor changes, which were noted for further during volunteer interviews. Minor amendments to the Gujarati LSAS were made, but one GP's suggestion for replacement of the phonetic translation of the word “diabetes” with the Gujarati term was not followed as it was felt that this might confuse people not familiar with the term.

Key aspects of the feedback were linked to perceptions of the purpose and usefulness of the LSAS and methods of encouraging its use. This qualitative feedback was received during pretest interviews with volunteers (stage 7); supporting quotations are provided as follows:

*Comprehension, Impact of the Risk Score, and Family History*

*It was easy. (Volunteer 13, male 35–60 yrs & Volunteer 14, male 35–60 yrs)*

*Whether you say risk or jokem it's the same thing. (Volunteer 10, male, aged over 60 yrs)*

*I need to work on my weight. It is a surprise, it is a surprise. Because I don't think that I would be on yellow level. I thought I was on green but I am on yellow so I need to work out for myself how to reduce my weight. I need to do some exercise to get my weight but I need less weight to come to the right group. Surprise yeah. I did not think I had any risk at all as no-one has diabetes in my family. My dad is 83 and he does not have anything. So surprise. (Volunteer 06, male, aged between 35–60 yrs)*

*It's helpful and very good. More than 75%, 80% is very good. The way in which people have explained, it will be helpful to Asians. Because it's about knowing what is going on within my body and I was able to think about it and that was helpful. (Volunteer 07, male, aged over 60 yrs)*

*It is helpful uh just to care if it's going to happen in the future I've got to be careful from the very beginning and take the precaution. (Volunteer 08, male, aged over 60 yrs)*

*I was shocked at the results um (pause) (Interviewer explored why)…mainly for myself by working out the tables and what they made me feel that I should do something for myself so it's that personal risk yeah….(Volunteer 18, female, aged between 35–60 yrs)*

*Using the Gujarati LSA in Different Settings*

*If it was in supermarkets it would be helpful to help people know where they stand. (Volunteer 11, male, aged over 60 yrs)*

*Online is really good but the people who are risk, the age range I believe, mainly 45+ or 50+ so majority of adults at that age. I must say I don't have a ratio of how many are IT literate and you know, so online is really good but there are certain issues whether they know how to operate computer whether they will be able to do it online. (Volunteer 02, female, aged between 35–60 yrs)*

*I think those that are interested in their health, and if it's in the mandir, then people may feel it's important because it's there. If we want to improve our life or take care of our body, if there is family history. (Volunteer 05, female, aged between 35–60 yrs)*

*Just giving out a leaflet like this would not work because these days people are lazy and do not want to read. […] but what you can do is give a lecture on this this and then give this out would be more helpful than just distributing all these things. (Volunteer 07, male aged over 60 yrs)*

It was evident from volunteers' responses that completion of the LSAS had impacted on a number of different levels, including awareness of preventative action and risk factors for T2DM such as weight. For a minority of volunteers, completion of the LSAS had also challenged beliefs that they were at low risk of developing T2DM because of a lack of family history of diabetes.

The suggestion of having the LSAS available online and in supermarkets and temples was discussed with most volunteers and a variation in views was apparent; some supported this idea and others doubted whether some people who are likely to be “at risk” due to age would be computer literate. A minority felt that only health conscious people would be interested in completing the LSAS in temples and supermarkets, but greater effectiveness might be achieved by providing a talk to accompany its distribution.

## 4. Discussion

In this paper, our findings have made a useful contribution to existing research by illustrating real world challenges to self-assessment of T2DM risk by non-English literate populations in the UK. We have shown that overall the translation model (Figure 1) was effective in achieving the study aims. The majority of the volunteers stated that they found the standard of Gujarati easy to read and understand. The model that we have developed is of significant relevance to healthcare researchers and commissioners internationally who wish to develop translated risk scores or other health assessment tools to meet the needs of populations speaking different languages.

Some aspects of the model used for developing the Gujarati version of the LSAS played key roles. Firstly, the preproject stage was not part of the formal development process but provided evidence of the need to undertake the project. In addition, this preliminary phase, involving feedback from focus groups with Gujarati and Punjabi participants, helped to inform a focused approach to the refinement of the original LSAS in English as the source document (Appendix 1). Secondly, additional stage 5 (Figure 1) was included, which shared some features associated with the committee approach [19] described in the literature. This entailed all four translators and project team working together to consider assumptions about terms, particularly those that had secondary meanings and dialectical differences. On reflection, this stage may have assisted in producing a better standard of translation and possibly reduced the time spent making changes in response to feedback from volunteers. It was noted, however, that this extra stage added to the duration and costs of the project.

Thirdly, the diversity of the translators' backgrounds and varied experience [14], knowledge of diabetes, and education (e.g., in terms of education within or outside of the UK) [19, 20] helped to produce a LSAS translation which was acceptable to a wide audience [15]. The need for such an approach was salient given the variation in the community of bilingual and monolingual readers of the target language. Additionally, aspects such as regional Indian dialects, mixing of Gujarati and English language in everyday use, “borrowing” of terms from other languages (such as East African languages) due to migration [15], and variations in educational levels further compound this variation. The sampling strategy for the pretest stage of the project enabled the project team to capture and take account of this variation in the level and standard of Gujarati used.

Lastly, eliciting responses and exploring volunteers' rational for these responses were possible through undertaking qualitative interviews for pretesting of the translated LSAS. The iterative process of making revisions and then conducting further interviews to test these changes allowed the project team to identify potential challenges to comprehension and respond rapidly and to test whether, for example, changes to the BMI table using systematic instructions were successful. This iterative approach also helped to identify the point at which no new major changes were required to the LSAS translation.

A frequently advocated approach to translation of research tools involves a process of decentring, when both the English and target language translations are developed concurrently [21]. Whilst this approach is resource intensive, its strength is the avoidance of translations situated in one culture [22]. Such an approach could be considered relevant; however, it was beyond the scope of this project as the English version of the LSAS is already widely used. Therefore, the project team were tasked with balancing the need to achieve conceptual equivalence of the English LSAS in Gujarati, without changing its construct [14]. Although revalidation of translated versions of instruments such as questionnaires is optimal, it was considered that our approach to obtaining equivalence in developing the Gujarati version of the LSAS would minimise any impact on the instrument's content validity and reliability, both of which had already been validated in the target population using the English version of the LSAS.

Outside the formal remit of the project, the Gujarati LSAS has been used at health fairs and informal feedback obtained has indicated that the language used is well understood. Completion of the LSAS and estimation of BMI were, however, still found to be challenging, suggesting the need for exploration of alternative means of calculation.

## 5. Conclusion 

Our experiences have drawn attention to challenges that are likely to be encountered in adapting a document of this type, as well as highlighting the overall usefulness of the model used. It is acknowledged that the translated version of the LSAS may require additional testing in other Gujarati speaking populations in the UK. The version developed was however found to be useful in facilitating wider access to the LSAS and promoting understanding of factors beyond family history when estimating personal risk of developing T2DM. Despite positive feedback regarding the translation, some operational problems still exist. Further development is required to allow calculation of BMI. In some cases support may need to be provided by people trained to use the LSAS. Providing this type of support as part of a risk self-assessment facilitated by community, faith, and voluntary organisations could ease the burden on the NHS and enhance the impact and reach of the NHS DPP in 2016.

## Supplementary Material

Appendix 1. Final English version of the Leicester Self Assessment Risk Score (LSAS) for type 2 diabetes used as the source document for translation.

## Figures and Tables

**Table 1:** An illustration of the challenges addressed in the translation with Gujarati words.

Terms and concepts	Challenge	How addressed
Risk and risk factors	A Gujarati equivalent that would convey the idea of risk in relation to diabetes was felt to be lacking; an appropriate Gujarati equivalent to the term risk factors was also felt to be lacking; initially, a phonetic translation of the English term was used, but some interview participants did not fully understand this	Initially, it was considered that a phonetic translation of the English word “risk” would be best, but many of the interview participants preferred the Gujarati word; in the final version, the initial paragraph used the phonetically spelt term “risk” with “jokhem” in brackets to help familiarise the reader, thereafter, throughout the document the Gujarati word for risk was used; an explanation of what risk factors are was provided, spelling “factors” phonetically and using the translated word for “reasons” rather than factors; this was tested on participants in the later interviews and it helped to aid comprehension

Ethnicity	Lack of equivalent Gujarati term to convey meaning	Rather than using a single word, a detailed explanation was given to aid conceptual and linguistic understanding and examples were given to aid comprehension- 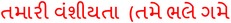 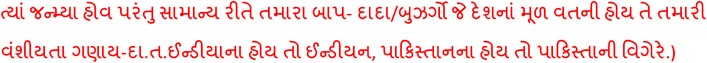

Lifestyle	Difficulty of conveying the intended meaning; some of the terms suggested by a minority of the interview participants had multiple meanings or were too general and did not capture the full meaning of the word life*style, *as opposed to *life*	We retained the translators' use of the Gujarati term for lifestyle 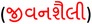 as participants understood the meaning

Questionnaire	Translation of the word questionnaire as *prashnavali* was perceived as being pitched at too high educational level by a minority of interview participants	A simple term used (*savalo ne yadi*- 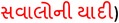

Term for diagnosis	A minority of interview participants suggested that the Gujarati word 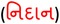 might be too technical, although they understood it themselves; discussion with the translators about other possible Gujarati equivalents suggested that these would give rise to ambiguity	It was decided to retain the original translation as this had been understood by interview participants and the translated version was aimed at people with a reading age of 12 or over

Terms used for “waist size group” (in the questionnaire) and “waist measurement” (in “how to measure your waist” instructions)	Some participants suggested using the Gujarati word for measurement (*map*) instead of waist size, acknowledging that it was not technically correct but commonly used and understood	After discussion, the word *map* 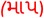 was used in the risk score and how to measure your waist instructions

The LSA states that…the good news is being diagnosed sooner rather than later…	The majority of interview participants found use of “good news” to be inappropriate and insensitive	This was revised to “it's good to know” 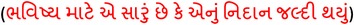 because you have been diagnosed early

Thrush (as a symptom of diabetes)	A detailed explanation of this term was deemed by the project team to be too technical, overly descriptive, and potentially distressing; the Gujarati word for thrush suggested by a few participants 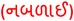 can also mean weakness and had the potential to create misunderstanding	The word for thrush 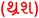 was phonetically translated in English

BMI table (the LSA includes a table for self-estimation of BMI)	The table was felt to be conceptually and practically challenging to use; the language used was not entirely the cause of the problem; providing examples did not appear to help with self-completion	This table was changed 3 times in response to feedback; what helped was simplifying the explanation and using systematic instructions similar to those used in for waist measurement; towards the end of the pretesting stage, it could be self-completed by some interview participants

## Additional Points

*Novelty Statement*. (i) The paper addresses a gap in published research evidence relating to perceptions and implications of using a diabetes risk score in people from black and minority ethnic backgrounds using the Gujarati language as an exemplar. (ii) We also describe and comment on the application of a cross-cultural translation model, based on methods commonly used to undertake cross-cultural translation of research instruments, to the translation of a diabetes risk score. (iii) The work described makes a timely contribution to understanding how the burden of risk assessment can potentially be eased in the national prevention programme for type 2 diabetes in the UK in 2016.
