# Supplementary material for: Developing a Conceptually Equivalent Type 2 Diabetes Risk Score for Indian Gujaratis in the UK
Source: J Diabetes Res. 2016 Sep 15;2016:8107108. doi: 10.1155/2016/8107108 (PMC5040802; doi:10.1155/2016/8107108)
Supplement: Supplementary file 1 — Appendix 1. Final English version of the Leicester Self Assessment Risk Score (LSAS) for type 2 diabetes used as the source document for translation. [file 8107108.f1.pdf]

# COULD **YOU** HAVE TYPE 2 DIABETES?

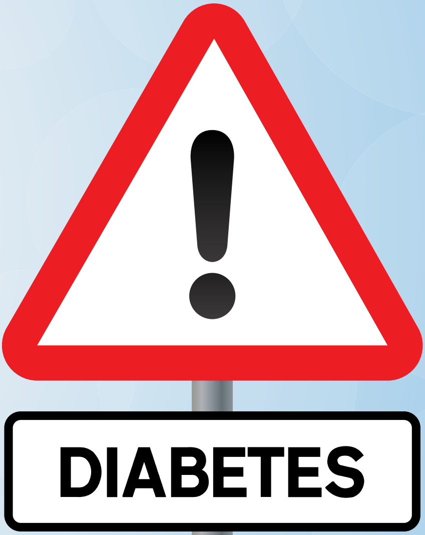


Type 2 diabetes develops when the body cannot control the amount of sugar in the blood. Type 2 diabetes can develop over a number of years without any symptoms. You can use this questionnaire to work out your own 'risk' of Type 2 diabetes.

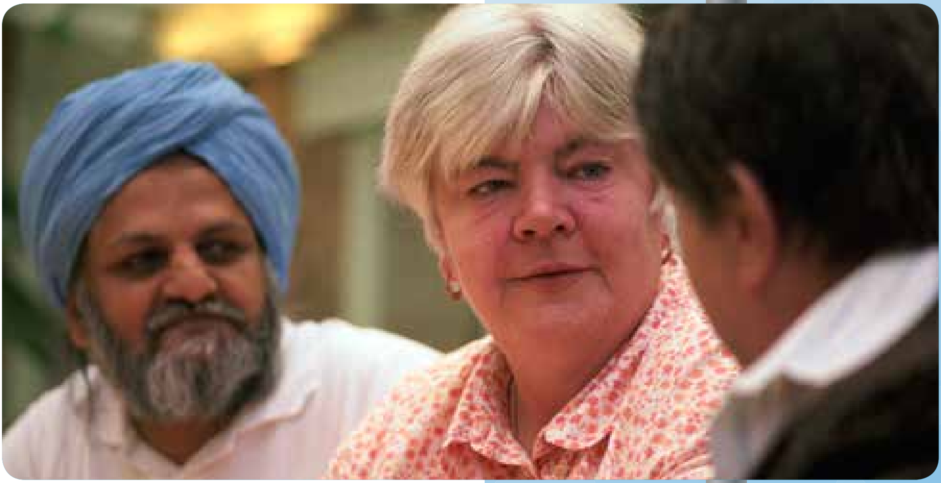

© Diabetes Research Centre - University of Leicester, 2016. Not to be reproduced whole or in part without the permission of the copyright owner.

© Diabetes Research Centre
University of Leicester, 2016.

## What does 'risk' mean?

'Risk' of Type 2 diabetes means how high is the chance of you having Type 2 diabetes now, or getting it in the future. Things which increase your risk of having Type 2 diabetes are called risk factors. Example risk factors are: being older, or having diabetes in your family.

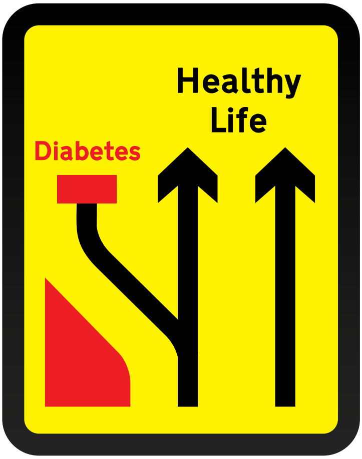


This questionnaire asks you about your risk factors to give you a 'risk score'.

## How will knowing your risk score help you?

Knowing your risk score will tell you what you need to do next. For some people, this may mean talking to their GP.

If you find out that you already have Type 2 diabetes, the good news is that being diagnosed sooner rather than later may help to prevent or delay future problems. You could reduce your chances of having heart attacks and strokes, and serious problems with your eyes, feet and kidneys.

If you find out that you have a higher risk of getting Type 2 diabetes in the future, there is some good news for you too. Making a few small changes (for example, to what you eat) can prevent or delay diabetes.

© Diabetes Research Centre
University of Leicester, 2016.

QUESTIONNAIRE: Do you want to know your risk of Type 2 diabetes?

For each question, tick one white box (✔).

| | | | | | |
|---|---|---|---|---|---|
| 1. Which age group are you in? | | | | | |
| 49 years and younger | | 0 | 60-69 years | | 9 |
| 50-59 years | | 5 | 70 years and/or older | | 13 |
| 2. Are you male or female? | | | | | |
| Male | | 1 | Female | | 0 |
| 3. How would you describe your ethnicity? | | | | | |
| White European | | 0 | Any other ethnic group | | 6 |
| 4. Do you have a parent, brother, sister and/or child with Type 1 or Type 2 diabetes? (Do not count step-relatives) | | | | | |
| Yes | | 5 | No | | 0 |
| 5. Which waist size group are you in? (See instructions on page 5) | | | | | |
| Less than 90 cm (35 inches) | | 0 | 100 -109 cm (39-42 inches) | | 6 |
| 90-99 cm (35-38 inches) | | 4 | 110 cm (43 inches) and above | | 9 |
| 6. Which Body Mass Index (BMI) group are you in? (See explanation and instructions on pages 6 and 7) | | | | | |
| Less than 25 | | 0 | 30-34 | | 5 |
| 25-29 | | 3 | 35+ | | 8 |
| 7. Have you ever been told by a doctor or nurse that you have high blood pressure? | | | | | |
| Yes | | 5 | No | | 0 |
| To get your risk score, add up the numbers in the blue boxes next to the seven boxes that you have ticked. Write the total number here – **This is your risk score:** To find out what this means go to pages 8 and 9 | | | | | |



© Diabetes Research Centre
University of Leicester, 2016.

## How to measure your waist

Measuring your waist with a tape measure is easy. The picture below will show you how to do it.

Get a tape measure.

1. Find your bottom rib
2. Find the top of your hip bone
3. Place the tape measure half way between your bottom rib and the top of your hip bone
4. Read your waist measurement to the nearest centimetre or inch. Go back to page 4 and complete question 5

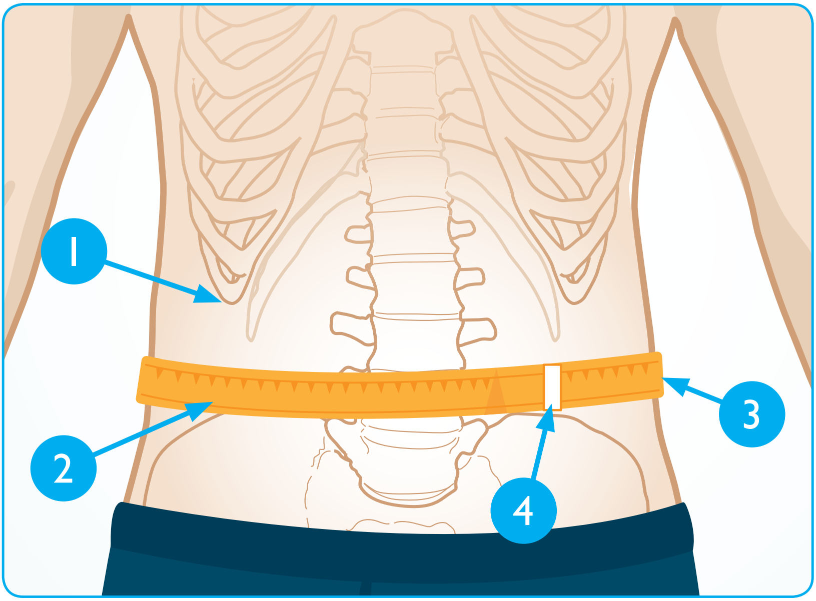


© Diabetes Research Centre
University of Leicester, 2016.

## Metric Body Mass Index (BMI)

Your BMI gives you a general idea of how healthy your weight is for your height. You can find out which BMI group you are in for your risk score by following the instructions below:

1. Find your height on the table in the left-hand column (blue)
2. Move along to the right of this column, to the box which states your weight
3. Then move down the column from this box to the coloured box at the end of the table, to find out your BMI group
4. Now, go back to the questionnaire on page 4, and tick the box for your BMI group on question 6

| Height (cm) | Weight (kg) | | | |
|---|---|---|---|---|
| 147 | Below 54 | 54-64 | 65-75 | 76+ |
| 150 | Below 57 | 57-67 | 68-78 | 79+ |
| 152 | Below 58 | 58-69 | 70-80 | 81+ |
| 155 | Below 60 | 60-71 | 72-83 | 84+ |
| 157 | Below 62 | 62-73 | 74-86 | 87+ |
| 160 | Below 64 | 64-76 | 77-89 | 90+ |
| 163 | Below 66 | 66-79 | 80-92 | 93+ |
| 165 | Below 68 | 68-81 | 82-95 | 96+ |
| 168 | Below 70 | 70-84 | 85-98 | 99+ |
| 170 | Below 73 | 73-86 | 87-101 | 102+ |
| 173 | Below 75 | 75-89 | 90-104 | 105+ |
| 175 | Below 77 | 77-91 | 92-107 | 108+ |
| 178 | Below 80 | 80-94 | 95-110 | 111+ |
| 180 | Below 81 | 81-97 | 98-113 | 114+ |
| 183 | Below 84 | 84-100 | 101-117 | 118+ |
| 185 | Below 86 | 86-102 | 103-119 | 120+ |
| 188 | Below 89 | 89-105 | 106-123 | 124+ |
| 191 | Below 92 | 92-109 | 110-127 | 128+ |
| 193 | Below 93 | 93-111 | 112-130 | 131+ |
| | Less than 25 | 25-29 | 30-34 | 35+ |



© Diabetes Research Centre
University of Leicester, 2016.

## Imperial Body Mass Index (BMI)

Your BMI gives you a general idea of how healthy your weight is for your height. You can find out which BMI group you are in for your risk score by following the instructions below:

1. Find your height on the table in the left-hand column (blue)
2. Move along to the right of this column, to the box which states your weight
3. Then move down the column from this box to the coloured box at the end of the table, to find out your BMI group
4. Now, go back to the questionnaire on page 4, and tick the box for your BMI group on question 6

| Height | Weight | | | |
|---|---|---|---|---|
| 4'8 | 7st 13 or less | 8st 0-9st 7 | 9st 8-11st 1 | 11st 2+ |
| 4'9 | 8st 3 or less | 8st 4-9st 12 | 9st 13-11st 7 | 11st 8+ |
| 4'10 | 8st 7 or less | 8st 8-10st 3 | 10st 4-11st 13 | 12st 0+ |
| 4'11 | 8st 11 or less | 8st 12-10st 8 | 10st 9-12st 5 | 12st 6+ |
| 5'0 | 9st 1 or less | 9st 2-10st 13 | 11st 0-12st 10 | 12st 11+ |
| 5'1 | 9st 6 or less | 9st 7-11st 4 | 11st 5-13st 2 | 13st 3+ |
| 5'2 | 9st 10 or less | 9st 11-11st 9 | 11st 10-13st 9 | 13st 10+ |
| 5'3 | 10st 0 or less | 10st 1-12st 1 | 12st 2-14st 1 | 14st 2+ |
| 5'4 | 10st 5 or less | 10st 6-12st 6 | 12st 7-14st 7 | 14st 8+ |
| 5'5 | 10st 9 or less | 10st 10-12st 11 | 12st 12-15st 0 | 15st 1+ |
| 5'6 | 11st 0 or less | 11st 1-13st 3 | 13st 4-15st 6 | 15st 7+ |
| 5'7 | 11st 5 or less | 11st 6-13st 9 | 13st 10-15st 13 | 16st 0+ |
| 5'8 | 11st 10 or less | 11st 11-14st 0 | 14st 1-16st 5 | 16st 6+ |
| 5'9 | 12st 0 or less | 12st 1-14st 6 | 14st 7-16st 12 | 16st 13+ |
| 5'10 | 12st 5 or less | 12st 6-14st 12 | 14st 13-17st 5 | 17st 6+ |
| 5'11 | 12st 10 or less | 12st 11-15st 4 | 15st 5-17st 12 | 17st 13+ |
| 6'0 | 13st 1 or less | 13st 2-15st 10 | 15st 11-18st 5 | 18st 6+ |
| 6'1 | 13st 7 or less | 13st 8-16st 3 | 16st 4-18st 12 | 18st 13+ |
| 6'2 | 13st 12 or less | 13st 13-16st 9 | 16st 10-19st 6 | 19st 7 + |
| | Less than 25 | 25-29 | 30-34 | 35+ |

© Diabetes Research Centre
University of Leicester, 2016.

## Your risk score

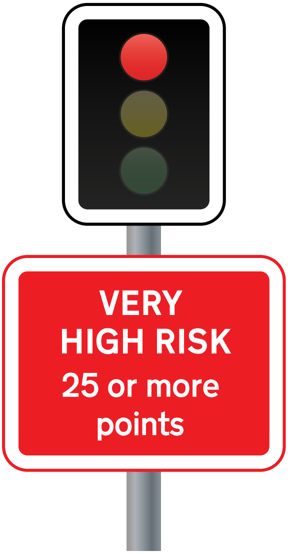


### What does this mean?

- 1 in 14 people in this group may have undiagnosed Type 2 diabetes
- 1 in 3 people in this group are at very high risk of developing Type 2 diabetes in the future

You have a **very high** chance of having Type 2 diabetes now or getting it in the future.

### What should you do now?

You need to visit your GP surgery for a diabetes test and to discuss your risk factors as soon as possible.

---

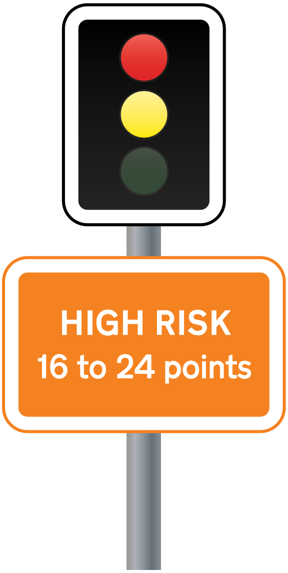


### What does this mean?

- 1 in 33 people in this group may have undiagnosed Type 2 diabetes
- 1 in 7 people in this group are at high risk of developing Type 2 diabetes in the future

You have a **high** chance of having Type 2 diabetes or getting it in the future.

### What should you do now?

You should discuss your risk score at your GP surgery; you may need a diabetes test. To reduce your risk, you should follow a healthy lifestyle by keeping active and eating a balanced diet. Take action to improve your lifestyle now!



© Diabetes Research Centre
University of Leicester, 2016.

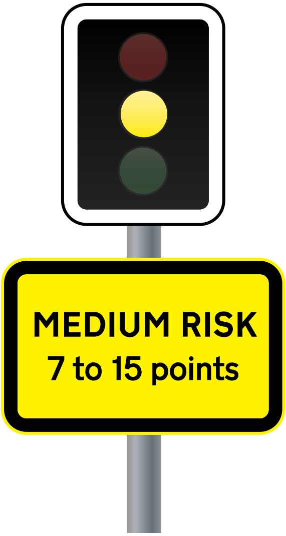


### What does this mean?

- 1 in 50 people in this group have may have undiagnosed Type 2 diabetes
- 1 in 10 people in this group are at high risk of developing Type 2 diabetes in the future

### What should you do now?

As you get older, your risk of developing Type 2 diabetes may increase. It is important that you follow a healthy lifestyle (keeping active and at a healthy weight) in order to reduce your risk of diabetes and other problems, such as heart disease or high blood pressure.

---

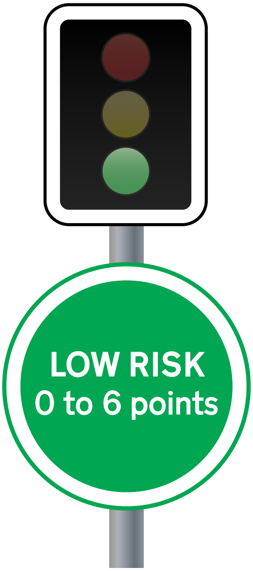


### What does this mean?

- 1 in 200 people in this group may have undiagnosed Type 2 diabetes
- 1 in 20 people in this group are at high risk of developing Type 2 diabetes in the future

### What should you do now?

As you get older, your risk of developing Type 2 diabetes may increase. It is important that you follow a healthy lifestyle (keeping active and at a healthy weight) in order to reduce your risk of diabetes and other problems such as heart disease or high blood pressure.

© Diabetes Research Centre
University of Leicester, 2016.

Whatever your risk score, you should go and see your GP or practice nurse immediately if you are experiencing any of the following symptoms:

- Feeling very thirsty
- Urinating more often than normal, particularly at night
- Extreme tiredness
- Blurred vision
- Weight loss and muscle pain
- Genital itching or regular episodes of thrush
- Slow healing of wounds

For more information or advice visit www.diabetes.org.uk or www.leicestershirediabetes.org.uk

## Disclaimer

We calculate your risk score using factors that our research indicates affects a person's likelihood of having undiagnosed Type 2 diabetes or impaired glucose regulation (which increases your likelihood of developing Type 2 diabetes in the future). Provided the information you input is accurate, the risk score should give a useful indication of the likelihood of having undiagnosed Type 2 diabetes or impaired glucose regulation. However, this is not a definitive test and should not be construed as medical advice or instruction. In any event, even where your risk score is low, there still remains an element of risk that you should be aware of.

The risk scoring tool and information here is of a general nature and is not intended to diagnose health problems nor replace the need for consulting a healthcare professional. No action should be taken based solely on the contents of this information; instead readers should consult appropriate medical professionals on any matter relating to their health.

We accept no responsibility for the use of the information provided.

If you have, or suspect you have, a health problem, you should consult a medical professional.



© Diabetes Research Centre
University of Leicester, 2016.

# Your Notes

© Diabetes Research Centre
University of Leicester, 2016.

Leicester Diabetes Centre
Leicester General Hospital
Gwendolen Road
Leicester LE5 4PW

www.leicesterdiabetescentre.org.uk
@LDC_Tweets
www.facebook.com/leicesterdiabetescentre

© Diabetes Research Centre University of Leicester, 2016. All rights reserved.
